# The possible onset of fibromyalgia following acute COVID-19 infection

**DOI:** 10.1371/journal.pone.0281593

**Published:** 2023-02-10

**Authors:** Einat Savin, Gali Rosenn, Avishai M. Tsur, Or Hen, Scott Ehrenberg, Omer Gendelman, Dan Buskila, Gilad Halpert, Daniela Amital, Howard Amital

**Affiliations:** 1 Department of Medicine ’B’, Zabludowicz Center for Autoimmune Diseases, Sheba Medical Center, Tel Hashomer, Ramat Gan, Israel; 2 Sackler Faculty of Medicine, Tel-Aviv University, Tel Aviv, Israel; 3 Israel Defense Forces, Medical Corps, Tel Hashomer, Ramat Gan, Israel; 4 Ben-Gurion University of the Negev, Beer Sheva, Israel; 5 Barzilai Medical Center, Ashkelon, Israel; Sant Anna Hospital: Clinica Sant’Anna, SWITZERLAND

## Abstract

**Introduction:**

The exact pathogenesis of fibromyalgia (FM) syndrome is unclear. However, various infectious have been implicated with the development of FM after their acute phase. We aimed to investigate the incidence of FM syndrome among convalesced individuals following hospitalization for Acute Coronavirus Disease-2019 (COVID-19).

**Methods:**

We performed a cross-sectional study on patients who were discharged after COVID-19 hospitalization from the Sheba Medical Center, Israel, between July 2020 to November 2020. A phone interview was performed consisting of the following questionnaires: the Fibromyalgia Survey Diagnostic Criteria Questionnaire, Sense of Coherence Questionnaire to evaluate resilience, and the Subjective Traumatic Outlook Questionnaire to assess the associated psychological aspects of the trauma. The incidence of post-COVID FM was calculated and regression models were performed to identify predictors.

**Results:**

The study population consisted of 198 eligible patients who completed the phone interview. The median age was 64 (52–72) and 37% were women. The median follow-up was 5.2 months (IQR 4.4–5.8). The incidence of FM was 15% (30 patients) and 87% (172 patients) had at least one FM-related symptom. Female gender was significantly associated with post-COVID FM (OR 3.65, p = 0.002). In addition, high median Subjective Traumatic Outlook scores and low median Sense of Coherence scores were both significantly associated with post-COVID FM (OR 1.19, p<0.001 and OR 0.92, p<0.001, respectively).

**Conclusions:**

FM is highly prevalent among COVID-19 convalescent patients. Our finding suggests that a significant subjective traumatic experience and a low resilience are highly associated with post-COVID FM.

## Introduction

Coronavirus Disease-2019 (COVID-19) is caused by the Severe Acute Respiratory Syndrome Coronavirus 2 (SARS-COV-2) and was declared as a global pandemic in early 2020 by the World Health Organization [1]. There is emerging evidence on the long-term symptoms and complications of COVID-19. Post-COVID, an evolving descriptive term of this syndrome, is defined as the persistence or development of new symptoms beyond four weeks from the onset of this disease [2]. The prevalent symptoms of post-COVID are fatigue, weakness, myalgia, arthralgia, cognitive impairment, sleep disorders, anxiety and depression [3,4].

Fibromyalgia (FM) syndrome is characterized by a chronic widespread musculoskeletal pain that frequently accompanied by fatigue, sleep, cognitive and mood disturbances [5]. The prevalence of FM ranges from 2 to 4% worldwide, with a female predominance, and it’s similar in different ethnic’s groups, cultures, and socio-economic classes [5–7]. The estimated prevalence of FM in the general Israeli population is 2.0–2.6%, similar to the observed in other Western countries [8]. Although the pathogenesis of FM is not well established, significant progress has been made during the recent years in clarifying the mechanism [9]. Stress has been shown to trigger FM, ranging from childhood neglect and emotional abuse [10], physical and sexual abuse [11], to the acute stress related to motor vehicle accident [12] and combat-related trauma [13].

We aimed to investigate the occurrence of the FM syndrome among convalesced individuals following hospitalization for Acute COVID-19 infection and to identify possible risk factors.

## Methods

### Study design and population

We performed a cross-sectional study that included all patients above 18 years old that had been discharged between July 15, 2020, to November 15, 2020, from the COVID-19 wards at Sheba Medical Center, Israel. All subjects had a confirmed positive SARS-COV-2 Polymerase Chain Reaction (PCR) test during their hospitalization. We excluded patients who were discharged to nursing homes or healthcare facilities, patients with pre-infection cognitive impairment, patients with an active psychotic disorder, patients with a prior diagnosis of FM, patients with a preexisting active malignancy, and patients who passed away during follow-up. A phone interview was conducted for eligible patients between January 19, 2021, and April 5, 2021, in chronological order according to the date of discharge.

The minimum interval time from discharge until the interview was 90 days. We retrieved from the electronic health record of Sheba Medical Center the demographic characteristics, medical history and the COVID-19 hospitalization details including severity, respiratory support, and the medications given. COVID-19 severity was classified, during the hospital stay according to the National Institutes of Health Criteria [14], ranging from mild (any symptoms without dyspnea or abnormal chest imaging), moderate (clinical or imaging evidence of lower respiratory disease with oxygen saturation ≥94%) and severe (patients with at least one of the following: oxygen saturation <94%, PaO2/FiO2 <300 mm Hg, respiratory frequency >30 breaths/min, or lung infiltrates >50% on chest imaging). The patients were discharged based on their clinical improvement. Negative laboratory PCR test was not necessary for discharge.

This study was approved by the ethics committee of the Sheba Medical Center, Israel (7577-20-SMC). All study participants granted informed consent prior to their inclusion in the study.

### Questionnaires

The phone interviewed included a series of questionnaires:

#### 1. The Fibromyalgia Survey Diagnostic Criteria Questionnaire

According to the 2010 American College of Rheumatology (ACR) Diagnostic Criteria (Modified 2011), the clinical criteria are based on the Widespread Pain Index (WPI), which includes 19 pain sites, and the Symptom Severity Score (SSS), that ranges from 0 to 12 [15]. This survey is considered positive for an FM diagnosis when the WPI is at least 7 with an SSS above 4, or when there is a WPI of 3–6 with an SSS score of 9 or higher, and there is no other disorder that could explain the symptoms. The Fibromyalgia Survey Diagnostic Criteria questionnaire was validated and was found to have good reliability, convergence and discriminant validity [16]. Moreover, the 2010 ACR modified 2011 was found to have higher sensitivity, specificity and correct classification compared with the ACR 2016 criteria [17].

#### 2. 13-items of the Antonovsky’s Sense Of Coherence (SOC) questionnaire

Two versions of the SOC scale were created by late Prof. Antonovsky. The first included 29 questions while the second included only 13 questions [18]. Both scales were found to be reliable, valid and cross-culturally applicable instruments in measuring a person capability to face stressful situations and remain well [19]. We chose the 13-iteams version because we found it more practical and easier to use. Each question is scored from 1 to 7, the scores of questions 1, 2, 3, 7, and 10 are inverted. The SOC score is the grand total points of the addition of each of the 13 questions’ respective point scores. According to the salutogenic model of health by Antonovsky [18], the SOC is a stable entity that forms in young adulthood [20], and it is a major individual resilience resource [21,22].

#### 3. 5-questions of the Subjective Traumatic Outlook (STO) questionnaire

The STO questionnaire was developed to assess individual differences in the way people subjectively perceive their trauma. Specifically, the STO is designed to evaluate the subject’s psychological condition prior to and following an event [23]. The validity of the questionnaire was tested among different Israeli populations and was found to have good convergent validity with similar, related subjective evaluations of post-traumatic stress disorder (PTSD) and PTSD-related constructs [23,24]. The STO may provide a useful screening tool to predict future risk for developing PTSD [24]. Each of the five questions are scored from 1 to 5 and the STO score is the grand total points of the addition of each of the 5 questions’ respective point scores. In 2019, recommended STO cut-off score were published for potential clinical use to measure PTSD and complex post-traumatic stress disorder (CPTSD) [25]. We used the PTSD algorithm, an STO Score of ≥ 13 indicates a high probability of progression to PTSD [25]. In our study, the participants were asked to refer to the COVID-19 acute phase as their traumatic-stress event.

The interview included the three questionnaires and was administrated by an internist. The physician was trained and practiced for the SOC and STO questionnaires by a psychiatrist, and for the fibromyalgia survey questionnaire by a rheumatologist, both highly experienced in the field of fibromyalgia.

### Statistical analysis

Post-COVID patients with and without fibromyalgia criteria were compared. Categorical variables were reported as n (%), and continuous variables were reported as the median and interquartile range (IQR). Chi-square, Fisher’s exact, and the Wilcoxon signed-rank tests were used appropriately to compare between the groups. Logistic regression models were used to determine the odds ratio (OR) and the 95% confidence interval (CI) between the SOC and STO scores, and the presence of FM. The multivariate model was adjusted for gender and COVID severity due to assumed associations to the scores and FM. All tests were two-tailed, with p values < 0.05 considered to be statistically significant. Data analysis was performed using R version 4.1.1 (R Core Team, Vienna, Austria).

## Results

**Fig 1:**
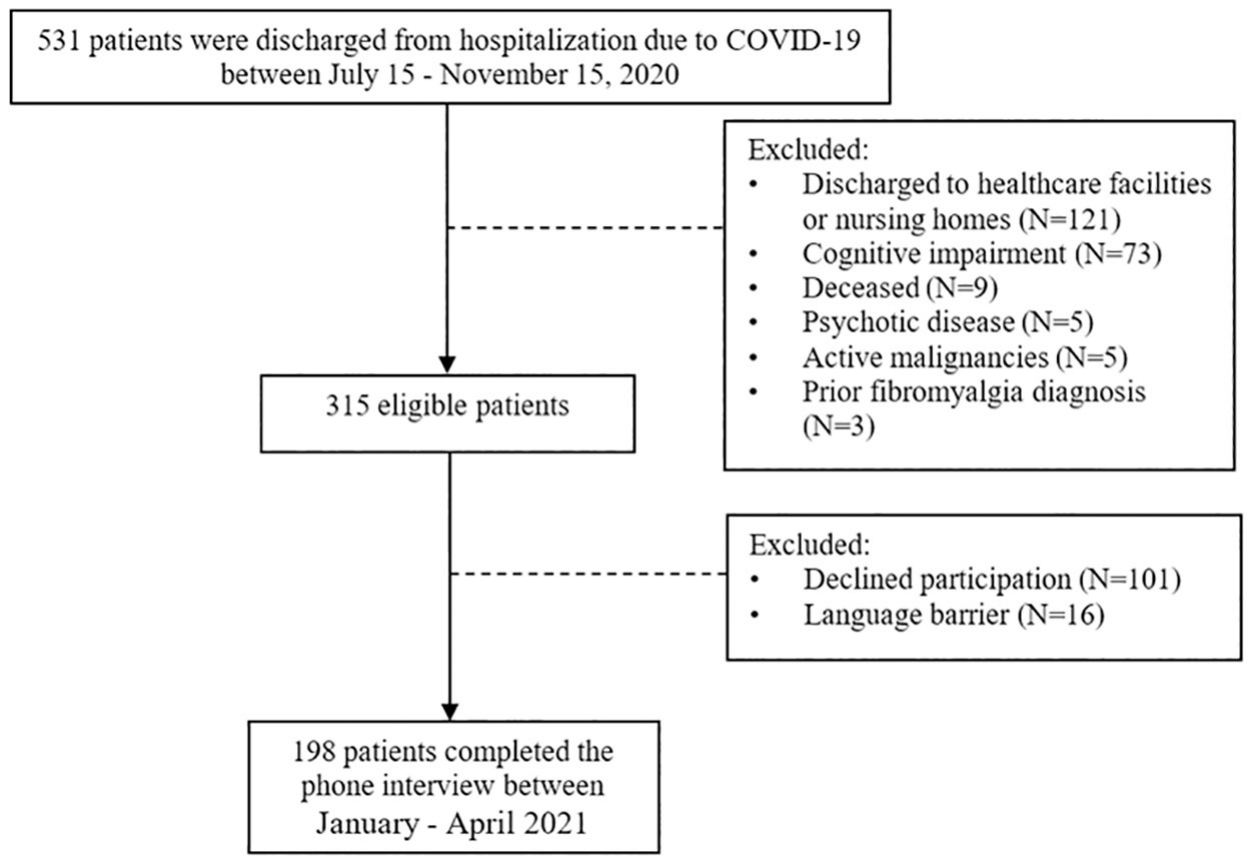
Flow diagram of patient selection.

A total of 531 patients were discharged from the COVID-19 wards at Sheba Medical Center between July 15, 2020 to November 15, 2020, and 216 were excluded according to above mentioned criteria. In addition, 101 patients declined participation and 16 had a language barrier, leaving 198 patients who completed the phone interview and were thereby included in the study (Fig 1). The baseline characteristics of the study population are presented in Table 1. The median time from discharge to phone interview was 5.2 months (IQR 4.4–5.8). The median age was 64 years (IQR 52–72), and 37% were women. Hypertension was the most common comorbidity (48%) followed by dyslipidemia (35%), diabetes mellitus (33%), obesity (22%), and congestive heart failure (19%). The acute phase of COVID-19 was severe in 59%, moderate in 10%, and mild in 31% of the study participants. A total of 30 patients (15%) fulfilled the criteria for FM diagnosis at follow-up, their characterstics are presented in Table 2. Moreover, 87% of the study population had at least one FM-related symptom according to the Fibromyalgia Survey Diagnostic Criteria questionnaire. The most common symptoms were fatigue (57%), unrefreshed sleep (56%), cognitive disturbances (54%) and muscle or joints pain (42%). In a univariate analysis, female gender was the only baseline factor predicting a post-COVID FM (OR 3.45, 95 CI 1.41–8.90, p = 0.008).

**Table 1:** Baseline characteristics of the study population.

Characteristic	Overall (n = 198)
Gender	
Men	125 (63%)
Women	73 (37%)
Age, years	64 (52–72)
Education	
High-school or lower	131 (66%)
Academic- Bachelor	51 (26%)
Master or higher	16 (8%)
Marital status	
Single	10 (5%)
Permanent relationship	150 (76%)
Divorced	22 (11%)
Widowed	16 (8%)
Comorbidity	
Hypertension	96 (48%)
Dyslipidemia	70 (35%)
Diabetes mellitus	65 (33%)
Obesity	43 (22%)
Congestive heart failure	38 (19%)
COVID-19 severity	
Mild	62 (31%)
Moderate	20 (10%)
Severe	116 (59%)
ICU admission	19 (9.6%)
Respiratory support during hospitalization	
Supplemental oxygen support	122 (62%)
NIV support	7 (3.5%)
HFNC support	37 (19%)
IMV support	3 (1.5%)
Treatments administration during hospitalization	
Corticosteroids	145 (73%)
Remdesivir	92 (46%)
Prophylactic LMWH	128 (65%)
Therapeutic dose of LMWH	57 (29%)
Length of hospitalization, days	4 (2–7)
Time from discharge to follow-up: Months	5.2 (4.4–5.8)

Data are n (%) or median (IQR). ICU = intensive care unit. HFNC = high-flow nasal cannula for oxygen therapy. NIV = non-invasive ventilation. IMV = invasive mechanical ventilation. LMWH = low molecular weight heparin.

**Table 2:** Baseline characterstics and questionnaires scores of patients with and without post-COVID Fibromylagia.

Characteristic	Fibromyalgia (n = 30)	Not fibromyalgia (n = 168)	p value
**Demographic factors**			
Age	60 (48–69)	65 (53–72)	0.15
Gender			
Woman	19 (63%)	54 (32%)	**0.001**
Education			0.3
High-school or lower	18 (60)	113 (67)	
Academic- Bachelor	11 (37)	40 (24)	
Master or higher	1 (3.3)	15 (8.9)	
Marital status			0.4
Single	0	10 (0.6)	
Permanent relationship	22 (73)	128 (76)	
Divorced	4 (13)	18 (11)	
Widowed	4 (13)	12 (7.1)	
**Clinical factors**			
Hypertension	12 (40%)	84 (50%)	0.3
Dyslipidemia	14 (47%)	56 (33%)	0.2
Diabetes mellitus	10 (33%)	55 (33%)	0.9
Obesity	7 (23%)	36 (21%)	0.8
Congestive heart failure	3 (10%)	35 (21%)	0.2
**COVID factors**			
Mild and Moderate COVID	14 (47%)	68 (40%)	0.5
Severe COVID	16 (53%)	100 (60%)	0.53
ICU admission	3 (10%)	16 (9.5%)	0.9
**Respiratory support during hospitalization**			
supplemental oxygen support	21 (70%)	101 (60%)	0.3
NIV support	3 (10%)	4 (2.4%)	0.072
HFNC support	5 (17%)	32 (19%)	0.8
IMV support	0 (0%)	3 (1.8%)	0.9
**Treatments administration during hospitalization**			
Treated with corticosteroids	21 (70%)	124 (74%)	0.7
Treated with Remdesivir	10 (33%)	82 (49%)	0.12
Treated with Prophylactic LMWH	20 (67%)	108 (64%)	0.8
Treated with Therapeutic dose of LMWH	10 (33%)	47 (28%)	0.6
Length of hospitalization, days	4.0 (1.2–7.8)	4.0 (2.0–7.0)	0.8
Length from discharge to follow up, days	156 (129–175)	159 (135–177)	0.5
**Questionnaires scores**			
Sense of coherence score			
Total score	67 (58–79)	83 (73–90)	**<0.001**
Comprehensibility	25 (21–29)	32 (28–35)	**<0.001**
Meaningfulness	19 (16–26)	26 (23–28)	**<0.001**
Manageability	22 (18–24)	26 (22–28)	**<0.001**
Subjective Traumatic Outlook score	19 (13–22)	7 (5–12)	**<0.001**

Data are n (%) or median (IQR). P value < 0.05 are bold. ICU = intensive care unit. HFNC = high-flow nasal cannula for oxygen therapy. NIV = non-invasive ventilation. IMV = invasive mechanical ventilation. LMWH = low molecular weight heparin.

COVID-19 acute phase severity, length of hospitalization and treatment regimens during hospitalization were not found to be significantly associated with a post-COVID FM (Table 3). Median STO score was higher in the FM group (19 vs.7, p value <0.001) while the SOC score was lower in the FM group compared with the non-FM group (67 vs. 83, p value <0.001). High STO and low SOC scores were found to be significantly associated with the existence of post-COVID FM (OR = 1.19 CI 95% 1.12–1.27 p<0.001, OR = 0.92 CI 95% 0.89–0.95 p<0.001, respectively). Adjusted multivariate logistic regression demonstrated that high STO and low SOC scores, as well as female gender, were found to be significantly associated with development of post-COVID FM (Table 3).

**Table 3:** Univariate and multivariate logistic regression of the association between patient’s characterstics, questionnaires scores and post-COVID Fibromyalgia.

	Univariarte			Multivariate		
Characteristic	Odds Ratio	95% Confidence interval	p value	Odds Ratio	95% Confidence interval	P value
**Demographic factors**						
Age	0.98	0.95–1.01	0.13	-	-	-
Woman gender	3.65	1.65–8.44	**0.002**	2.88	1.12–7.81	**0.031**
**Clinical factors**						
Hypertension comorbidity	0.67	0.30–1.46	0.31	-	-	-
Dyslipidemia comorbidity	1.75	0.79–3.85	0.16	-	-	-
Diabetes mellitus comorbidity	1.03	0.43–2.30	0.95	-	-	-
Obesity comorbidity	1.12	0.42–2.70	0.82	-	-	-
Congestive heart failure comorbidity	0.42	0.10–1.29	0.18	-	-	-
**COVID factors**						
Severe COVID	0.78	0.36–1.71	0.53	0.54	0.20–1.41	0.2
supplemental oxygen support	1.55	0.69–3.75	0.31	-	-	-
NIV support	4.56	0.86–21.7	0.055	-	-	-
HFNC support	0.85	0.27–2.23	0.76	-	-	-
Treated with corticosteroids	0.83	0.36–2.03	0.66	-	-	-
Treated with Remdesivir	0.52	0.22–1.16	0.12	-	-	-
Treated with Prophylactic LMWH	1.11	0.50–2.62	0.8	-	-	-
Treated with Therapeutic dose of LMWH	1.29	0.54–2.90	0.55	-	-	-
Length of hospitalization	1.01	0.93–1.08	0.79	-	-	-
Length from discharge to follow-up	0.99	0.98–1.01	0.33	-	-	-
**Questionnaires scores**						
Sense of coherence						
Total score	0.92	0.89–0.95	**<0.001**	0.76	0.63–0.90	**0.002**
Comprehensibility	0.87	0.81–0.92	**<0.001**	-	-	-
Meaningfulness	0.82	0.75–0.88	**<0.001**	-	-	-
Manageability	0.84	0.78–0.91	**<0.001**	-	-	-
Subjective Traumatic Outlook	1.19	1.12–1.27	**<0.001**	1.81	1.26–2.66	**0.002**

P value < 0.05 are bold. HFNC = high-flow nasal cannula for oxygen therapy.

NIV = non-invasive ventilation. IMV = invasive mechanical ventilation. LMWH = low molecular weight heparin.

## Discussion

Long-term consequences of COVID-19 have recently led to emerging research interests [3,4], probably due to their influences of the psychiatric, functional, and occupational states of theses convalesced patients as well as their impact on public health expenditure [26]. Different infections including hepatitis C virus, Human immunodeficiency virus and Lyme disease have already been implicated with the development of FM after their acute phase [27]. Imbalance between pro-inflammatory and anti-inflammatory cytokines has been suggested as a possible mechanism that facilitates the neuropathic pain [28].

In this study we have demonstrated that at five months following hospitalization for Acute COVID-19 infection, 15% of the patients and 26% of the women fulfilled the FM diagnostic criteria, approximately five times the proportion of FM in the Israeli general population [6,8]. In addition, most of these investigated patients had at least one FM-related symptom at 5 months follow-up.

Similarly to our results, a recent study by Ursini et al. [29], based on a self-internet survey that was distributed on social networking sites, reported that 30% of 616 individuals who were recovered from COVID-19 infection fulfilled the criteria for FM. The difference with our results in their reported percentage may attributed to a selection bias in their study. In our opinion, the difference with our results in their reported percentage may be attributed to a selection bias. Higher rate of women responders (77.4%) compared with ours (37%), may be due to the well-known gender bias favoring women in survey-based research [30]. Our study design attempted to reduce this bias. Initially, by selection of the entire population of COVID-19 patients that were hospitalized in our departments and by a telephone contact which was shown to raise the response rates by 10% [31]. Our results are also consistent with previous studies which investigated the long-term symptoms of Post COVID-19 and demonstrated that most of these patients continued to suffer from at least one FM-related symptom such as fatigue (35–63%), myalgia (20–48%), sleep disorders (26–47%), concentration disturbances (28%) and anxiety or depression (23%) for a period ranging from 3 to 12 months following the infection [3,4,32,33]. Consistent with our results, women had a higher prevalence of post-COVID symptoms as has been reported by Lombardo et al. [4] and Huang et al. [3] (81% vs. 77% p = 0.02, and 81% vs. 73% p<0.01 respectively). In addition, we didn’t find a significant association between the occurrence of post-COVID FM and the COVID-19 acute phase severity or the treatment regimen utilized, which has been confirmed in a previous investigation [4].

In our study, the median Subjective Traumatic Outlook score was 8 (IQR 5–14), and 73% of the FM group had a Subjective Traumatic Outlook score ≥ 13 compared with 22% in the non-FM group. Psychosocial and mental effects due to acute COVID-19 disease have been reported in a few previous studies. In one investigation, PTSD was observed in 22.2% of 185 COVID-19 convalesced patients almost one month after discharge, and female gender was significantly associated with PTSD development (OR, 4.03, 95% CI 1.76 to 9.47, p = 0.0011) [34]. In another study, approximately 33% of 100 COVID-19 convalesced patients had at least one PTSD-related symptom 4–8 weeks after discharge [35]. In the development of FM, a matrix of interaction between genetic factors, and life-long environmental exposures plays a role. The association between physical and psychological trauma have been described in the medical literature as preceding and even triggering the development of FM. Triggers such as physical assault, motor vehicle accident, emotional anguish and acute illness were found to be highly associated with development of FM [11,36]. Our findings link FM symptoms with both physical stress due to the acute infection as well as the traumatic inpatient experience among patients who were hospitalized as a result of COVID-19 infection in 2020 [37].

A further interesting finding in our study is the significant correlation between a lower Sense of Coherence and the incidence of post-COVID FM (OR = 0.92, CI 95% 0.89–0.95, p value <0.001). In a meta-analysis by Schäfer et al. [38] consisting of 45 studies and 10,883 participants, revealed a substantial correlation between a low Sense of Coherence level and PTSD symptom severity (mean *M*(*r*) = −.41). We suggest that poor resilience might be related to the pathogenesis of FM following Acute COVID-19 infection, and that poor resilience in FM patients may explain their relatively high prevalence of PTSD symptoms [39].

The current study is only the second one that investigated the occurrence of FM following Acute COVID-19 infection, and the first to have investigated occurrence of FM after hospitalization due to COVID-19 infection. This study is also the first investigation that attempts to predict the risk factors for developing FM following Acute COVID-19 infection.

Our study has several limitations. Firstly, there was a relatively small number of participants, and there was no control group. Second, there was no baseline questionnaires recorded before our patients developed their Acute COVID-19 infection. Third, although this Fibromyalgia Survey Diagnostic Criteria Questionnaire has been previously validated and was found to have good reliability, convergence and discriminant validity [16], the FM symptoms were evaluated subjectively, and not objectively by physical exam. Finally, the SOC and STO questionnaires were conducted by an internist that was trained by psychiatrist.

## Conclusions

Our findings suggest that Acute COVID-19 convalesced individuals have a higher proportion of FM compared to the general population. The presence of a female gender, low resilience and a significant subjective traumatic experience were found to be significantly associated with the occurrence of post-COVID FM.
